# An Update for Pharmacologists on New Treatment Options for Inflammatory Bowel Disease: The Clinicians’ Perspective

**DOI:** 10.3389/fphar.2021.655054

**Published:** 2021-04-12

**Authors:** Carsten Schmidt, Philip C. Grunert, Andreas Stallmach

**Affiliations:** ^1^ Medizinische Klinik II, Klinikum Fulda AG, Universitätsmedizin Marburg - Campus Fulda, Fulda, Germany; ^2^ Klinik für Innere Medizin IV, Universitätsklinikum Jena, Friedrich-Schiller Universität Jena, Jena, Germany

**Keywords:** interleukin-23, anti-integrin drugs, sphingosine-1-phosphate receptor modulator, JAK inhibitor, fecal microbiota transplantation, biologics, small molecule

## Abstract

The introduction of anti-tumor necrosis factor antibodies resulted in a considerable expansion of the options available for the treatment of inflammatory bowel disease. Unfortunately, approximately one third of treated patients do not respond to these modalities, and drug efficacy may be lost over time. These drugs are also associated with contraindications, adverse events, and intolerance. As such, there is an ongoing need for new therapeutic strategies. Despite several recent advances, including antibodies against pro-inflammatory cytokines and cell adhesion molecules, Janus kinase inhibitors, and modulators of sphingosine-1-phosphate receptors, not all problems associated with IBD have been solved. In this manuscript, we review the current state of development of several new treatment options. Ongoing evaluation will require specific proof of efficacy as well as direct comparisons with established treatments. Results from head-to-head comparisons are needed to provide clinicians with critical information on how to formulate effective therapeutic approaches for each patient.

## Introduction

Patients diagnosed with inflammatory bowel disease (IBD), including those with Crohn´s disease (CD) and ulcerative colitis (UC), can be difficult to manage clinically given the broad spectrum of disease, including both intestinal and extraintestinal manifestations. IBD is an immunologically-mediated disease with increasing prevalence across Western Europe, North America, and Australia, as well as in newly-industrialized areas of Asia and South America. The prevalence of IBD has been estimated at 0.3–0.5% in Western Europe, including at least 420,000 patients in Germany alone. Similarly, population-based data from Canada predict that the prevalence of IBD, estimated at 725 patients per 100,000 (0.73%) in 2018 will increase to as many as 981 patients per 100,000 (0.98%) in 2030 (Coward et al., 2019). If we apply this calculation to the current patient population in Germany, we might anticipate an increase to 0.67% in 2030, representing >560,000 patients.

While the physiologic mechanisms contributing to the development of IBD have not been fully clarified, current research suggests that genetically-susceptible individuals respond to routine environmental factors with alterations at the gastrointestinal barrier and exaggerated (or poorly-suppressed) innate and adaptive immune responses. These findings have provided a theoretical and practical basis for the development of new therapies. Indeed, the introduction of the anti-tumor necrosis factor (TNF) antibody, infliximab, first for CD (in 1999) and later for UC (in 2006), revolutionized the treatment of these conditions. Other anti-TNF antibodies (all under the broad category known as “biologics”), including adalimumab, golimumab, or certolizumab pegol [certolizumab pegol is not approved by the European Medicines Agency (EMA)] were developed shortly thereafter. With the availability of biosimilars for infliximab and adalimumab and the approval of both ustekinumab, a monoclonal antibody (mAb) that antagonizes the actions of proinflammatory cytokines IL-12 and IL-23, and vedolizumab, a mAb that specifically targets α_4_β_7_-integrin, physicians now have access to a broad range of unique biologics that can be used to manage intestinal inflammation. This armamentarium also includes tofacitinib, which is a small-molecule inhibitor of Janus kinases (JAKs) that can be used to treat patients diagnosed with UC. Unfortunately, not all patients respond to these new therapies (known as “primary non-responders”). Depending on the respective phase III study, the proportion of primary non-responders may be as high as 30–50%. Other patients may experience secondary loss of response due to the development of neutralizing antibodies or *via* other, as yet not well-understood mechanisms (e.g., as observed in patients treated with tofacitinib). The comparatively high incidence of adverse events also limits the use of these new therapeutic options. As such, additional research is needed that focuses on approaches that can be used to achieve long-lasting, steroid-free remission without adverse effects in all patients diagnosed with IBD.

**FIGURE 1:**
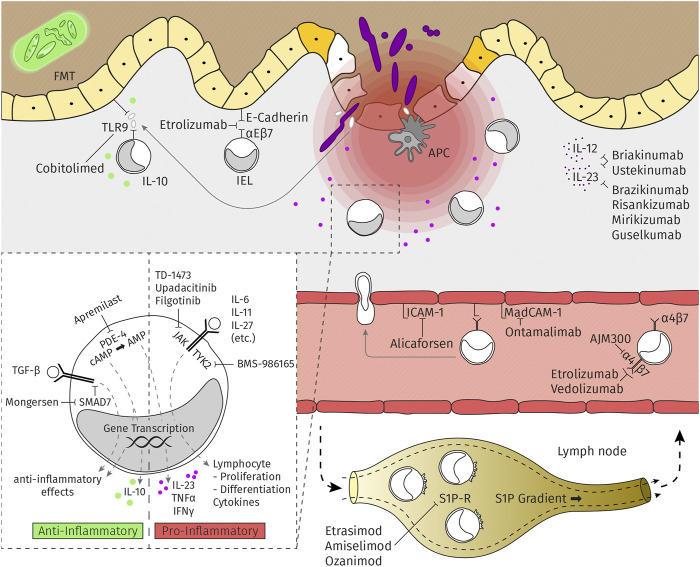
Mechanisms of action of several of the new therapeutic agents used to treat IBD.

The mechanisms of action of the new therapeutic agents used to treat IBD are summarized in Figure 1. It is critical to understand that the published studies focused on each of these agents feature different patient populations and have different inclusion and exclusion criteria; among these are differences in disease duration and activity, previous experience with anti-IBD therapies, duration of induction therapy, primary endpoints, and design of maintenance protocols (i.e., “treat-through” vs. “randomized responder”). As such, significant caution must be exercised when comparing the results of different studies.

Due to the multitude of new as well as already established modes of action and specific substances for the treatment of IBD a targeted selection of a medication for an individual patient is of utmost importance. Therefore, several biomarkers (e.g., fecal calprotectin, CRP, serological and genetic parameters, histological findings and microbiota) have been and are currently being evaluated with regard to the prediction of treatment response. At present, however, biomarkers of this type are not yet established for daily clinical practice. Further, from a clinical point of view improvement of disease activity and patient related outcome parameters are more important than changes in biomarkers. For these reasons, we aimed not to discuss such parameters in this overview on the effectiveness and safety of new therapeutics.

An electronic database search using PubMed, spanning up to November 2020, was conducted. Abstracts were also reviewed from Digestive Diseases Week, European Crohn’s and Colitis Organization congress, and United European Gastroenterology Week 2019 and 2020, respectively.

## Anti-Integrin Antibodies

Integrins are cell adhesion molecules that form transmembrane dimers consisting of *α*- and *β*-chains. The goal of an anti-integrin strategy is to block the actions of adhesion molecules on circulating immune cells and/or those of their receptors on endothelial cells. For example, *α*_4_β_7_-integrin on the surface of CD4^+^ T lymphocytes binds specifically to MadCAM-1 (mucosal addressin cell adhesion molecule-1) on endothelial cells in the gastrointestinal tract and thereby mediates “gut-selective” lymphocyte migration into the gastrointestinal mucosa. **Vedolizumab** is a humanized mAb that targets *α*_4_β_7_-integrin and was first approved for the treatment of CD and UC in 2014 with a favorable safety profile in clinical trials. This assessment was reinforced with the first direct, head-to-head comparison of biologic therapies for IBD carried out in 2019 as the VARSITY study. The results of this study revealed that vedolizumab had a more favorable therapeutic impact on inducing clinical remission compared to results obtained using adalimumab (Sands et al., 2019a).

Given the overall efficacy and “gut-selective” effects of vedolizumab, it is not surprising that cell adhesion molecules are among the most prominent targets of new therapeutics under development. Various inhibitors of the integrin *β*_7_-subunit and its endothelial ligand, MadCAM-1, are currently undergoing evaluation in numerous studies. For example, in one phase II study, Vermeire and colleagues (Vermeire et al., 2014) found that the anti-*β*_7_ antibody, **etrolizumab**, was more effective than placebo at inducing remission in patients with symptomatic UC. One important aspect of etrolizumab is the fact that it targets not only *α*_4_β_7_-integrin (similar to vedolizumab) but also can inhibit α_E_β_7_-integrin-mediated interactions between intraepithelial lymphocytes and E-cadherin expressed by enterocytes, thereby reducing the extent of lymphocyte accumulation at this site. This is a critical finding, as the probability of remission correlated strongly with mucosal expression of the *α*_E_-subunit, which was also identified as the first established predictor of the response to mAb therapy. These results were followed by eight randomized-controlled and open-label studies of this modality for the treatment of CD and UC. More than 3,000 UC and CD patients were enrolled in two open-label and safety studies as well as in six phase III trials, including comparative studies against both adalimumab and infliximab (Sandborn et al., 2020e). However, in August 2020, Roche investigators reported mixed results from their studies and announced that the study program for patients diagnosed with UC was to be halted, while studies focused on CD were to be continued. The favorable safety profile for etrolizumab was consistent with the results of previous studies, although the results of the various treatment studies for UC were not as convincing. In the “Hibiscus-I” induction study, the primary endpoint of clinical remission at week 10 was achieved in response to etrolizumab among patients who had not undergone prior treatment with anti-TNF mAbs (19.4 vs. 6.9%; *p* = 0.0173). By contrast, in the “*Hibiscus* II” induction study (which also included anti-TNF-naïve patients) the same primary endpoint was not met (18.2 vs. 11.1%; *p* = 0.1729) (Dotan et al., 2020). In the “Hickory” study, the primary endpoint of induction (clinical remission at week 14: 18.5 vs. 6.3%; *p* = 0.0033), but not maintenance of remission (among clinical responders at week 14) was achieved in response to etrolizumab among patients with a history of previous anti-TNF treatment (24.1 vs. 20.2% at week 66; *p* = 0.4956) (Peyrin-Biroulet et al., 2020a). Finally, in the “Laurel” maintenance study, the primary endpoint of clinical remission at week 62 among responders at week 10 was not achieved in patients with no history of anti-TNF treatment (29.6 vs. 20.6%; *p* = 0.1942) (Vermeire et al., 2020). The consequences of these results and their impact on the further development of etrolizumab remain to be determined.

**AJM300** is a small molecule administered orally that blocks the *α*_4_-integrin subunit that has been investigated in patients with moderately-active UC. In one phase II study, 102 patients were treated with 960 mg AJM300 or placebo three times a day for 8 weeks. 62.7% of the patients in the treatment (AJM300) group exhibited a clinical response (a decrease in the Mayo Score by >3 points or at least 30% with a reduction in the rectal bleeding subscore by >1 point) compared to only 25.5% of the patients in the placebo group (*p* = 0.002). Furthermore, clinical remission was observed in 23.5% vs. 3.9% of patients (*p* = 0.01) (Yoshimura et al., 2015); This drug is currently undergoing evaluation in a phase III study involving patients with UC.

**Ontamalimab (PF-00547659)** is a mAb that targets MadCAM-1. A phase II study in patients with UC documented the superiority of this drug in inducing clinical remission compared to placebo (16.7% vs. 2.7% at week 12; *p* = 0.01). Patients who responded to therapy were treated for up to 72 weeks in an open-label extension study; in this study, positive effects on the maintenance of remission were more pronounced in patients that received higher drug doses. By contrast, no differences were observed in a comparison between PF-00547659 and placebo in patients with moderate-to-severe CD, despite the observed decreases in the concentration of circulating MAdCAM-1 and a dose-dependent increase in β7^+^ memory T-cells, which are both findings that document the pharmacologic efficacy of the drug (Sandborn et al., 2018). Takeda (who acquired ontamalimab through the acquisition of Shire in 2018) announced the end of the study program in May 2020.

The results of six studies focused on achieving clinical remission in both CD and UC using anti-integrin strategies are summarized in Table 1.

**TABLE 1:** Frequency of achieving clinical remission in anti-integrin directed studies of patients with CD (a) and UC (b).

Drug	Study phase	Induction treatment				Study type	Maintenance treatment				References
		Week	Study drug	Placebo	*p*		Week	Study drug	Placebo	*p*	
a) CD											
Vedolizumab^a^	III	6	14.5	6.8%	0.02	rr	85	39.0	21.6%	<0.001	Sandborn et al. (2013)
Ontamalimab	II	8	29.1	16.7%	n.s						Sandborn et al. (2018)

Drug	Study phase	Induction treatment				Study type	Maintenance treatment				References
		Week	Study drug	Placebo	*p*		Week	Study drug	Placebo	*p*	
b) UC											
Vedolizumab^a^	III	6	16.9	5.4%	<0.001	rr	52	44.8	15.9%	<0.001	Feagan et al. (2013)
Etrolizumab	III	10	19.4	6.9%	0.0173	rr	62	29.6	20.6%	0,194	Dotan et al. (2020)
	III	10	18.2	11.1%	0.1729						Vermeire et al. (2020)
Etrolizumab	III	14	18.5	6.3%	0.0033	rr	66	24.1	20.2%	0,4956	Peyrin-Biroulet et al. (2020a)
Ontamalimab	II	12	16.7	2.7%	0.01	OLE	40–72	22.8	nd	Na	Vermeire et al. (2017a), Danese et al. (2019)
AJM300	II	8	23.5	3.9%	0.01						Yoshimura et al. (2015)

NB: In studies that feature different dosages or intervals, the most effective treatment has been presented.

^a^ currently licensed for use as therapy in CD and UC; OLE, open-label extension; tt, treat-through; r, continuation of assigned treatment in responders; r 26, remission at week 26; rr, randomized responder; nd, not done; na, not applicable.

## Comment

Given the well-characterized positive responses to treatment with vedolizumab, the largely negative results of studies focused on etrolizumab for the treatment of UC are somewhat surprising. The full publications may ultimately provide more information to explain the underlying issues. Given the well-established favorable safety profile for vedolizumab, therapeutic agents directed against integrin-mediated interactions might be suitable for use as combination therapy with drugs that have different mechanisms of action. Results of phase III studies of etrolizumab combined with other therapies may provide further insight into this possibility.

## Blockade of Interleukin-23

Interleukin-12 (IL-12) and interleukin-23 (IL-23) are heterodimeric cytokines that share a common p40 subunit; this subunit has been identified as an effective target for mAbs designed to inhibit the actions of both pro-inflammatory mediators. By contrast, mAbs that target the second subunit of IL-23 (p19) alone promote selective inhibition of this cytokine. The anti-p40 mAb, **ustekinumab,** was approved for use in 2016, has proven effectiveness and a good safety profile when used to treat patients with CD and UC (Feagan et al., 2016; Sands et al., 2019b).

**Briakinumab** is another anti-p40 mAb that is currently under study for the treatment of psoriasis. Although higher response and remission rates were observed at weeks 6, 12, and 24 in a phase IIb study of patients with CD, the primary endpoint of the study, clinical remission at week 6, was not achieved (13,7 vs. 8.7%; *p* = 0.157) (Panaccione et al., 2015).

The potential use of **brazikumab (MEDI2070)**, a selective anti-p19mAb, was evaluated in a phase IIa study. In this trial, 119 patients with CD who had failed previous treatment with anti-TNF antibodies achieved a clinical response to brazikumab after 8 weeks, at a significantly higher rate than was observed for patients in the placebo-control group (49.2 vs. 26.7%; *p* = 0.01). Interestingly, higher serum concentrations of interleukin 22 (a “downstream” mediator in the IL-23 signaling pathway) were positively associated with treatment response; as such, IL-22 levels may be a predictor of treatment response (Sands et al., 2017).

Similarly, results of a phase II trial involving **risankizumab**, another anti-p19 mAb, revealed clinical remission at 12 weeks in 121 patients with CD significantly more frequently than in those receiving placebo (36.6 vs. 15.4%; *p* = 0.0252) (Feagan et al., 2017). When therapy was continued in the subset of patients who had achieved remission at week 26, clinical and endoscopic remission was achieved in 71 and 55% of the patients examined at week 52, respectively (Feagan et al., 2018).

In a phase II study of the anti-p19 mAb, **mirikizumab**, in 168 patients with UC, clinical remission was observed significantly more frequently in patients who received the study drug than in those receiving placebo (22.6 vs. 4.8%; *p* = 0.004), In this study, clinical responses were observed after intravenous induction therapy at a dose of 200 mg at weeks 0, 4 and 8; however, as insufficient responses were observed to doses of 50 or 600 mg, the primary endpoint of the study was not met. Nonetheless, it is important to note that clinical responses to therapy were observed more frequently among those who received one of the two higher doses (60 and 49% to 200 and 600 mg, respectively) than those in the placebo group (21%). Upon continuation of therapy, clinical remission was maintained up to week 52 in 37–47% of patients, depending on the dosage interval. As such, any future studies must first address the issue of the optimal dosage for induction therapy (Sandborn et al., 2020b).

In another phase II study featuring mirikizumab carried out in 191 CD patients, an endoscopic response (primary endpoint) was achieved significantly more frequently with induction doses of 600 mg (37.5%; *p* = 0.03) or 1,000 mg (43.8%; *p* < 0.001) than with placebo (10.9%). The rate of clinical response and clinical remission [600 mg: 40.6% (*p* < 0.001), 1,000 mg: 26.6% (*p* = 0.013) vs. placebo (9.4%), respectively] was also achieved significantly more frequently with mirikizumab (Sands et al., 2019). Findings in a recently published abstract reported that maintenance therapy after successful induction of an endoscopic response was successful in 59% of patients up to week 52, while clinical remission was achieved in 46% of patients (Sands et al., 2020a). A phase III study program (VIVID) aimed at evaluating the clinical efficacy of mirikizumab for the treatment of patients with CD is currently underway.

At United European Gastroenterology (UEG) Week 2020, data from the GALAXI 1 study that featured the treatment of CD patients with the anti-p19 mAb, **guselkumab,** were presented. Similar to risankizumab, this drug has already been approved for the treatment of plaque psoriasis. In this phase II study, the effectiveness of intravenous induction therapy with guselkumab was compared to placebo and induction with ustekinumab in 250 patients. Clinical remission was observed in 50–56% of patients at week 12 (and in 16 and 45% to placebo and ustekinumab, respectively). Other endpoints, including clinical, biochemical, and endoscopic responses were also achieved significantly more frequently with guselkumab than with placebo, although no dose-dependency was observed (Sandborn et al., 2020).

The results of eight studies focused on achieving clinical remission in both CD and UC using anti-IL-23 strategies are summarized in Table 2.

**TABLE 2:** Frequency of achieving clinical remission in anti-IL-23 (±anti-IL-12) directed studies of patients with CD (a) or UC (b).

Drug	Study phase	Induction treatment				Study type	Maintenance treatment				References
		Week	Study drug	Placebo	*p*		Week	Study drug	Placebo	*p*	
a) CD											
Ustekinumab^a^	III	8	29.8	13.0%	<0.001	rr	52	53.1	35.9%	0.005	Feagan et al. (2016)
Briakinumab	II	6	17.3	8.7%	0.157						Panaccione et al. (2015)
Risankizumab	II	12	36.6	15.4%	0.0252	r 26	52	71.0	nd	Na	Feagan et al. (2017), Feagan et al. (2018)
Mirikizumab	II	12	40.6	9.4%	<0.001	rr	52	56.5	nd	Na	Sands et al. (2019), Sands et al. (2020b)
Brazikumab	II	8	27.1	15.0%	0.1						Sands et al. (2017)
Guselkumab	II	12	56.0	15.7%	0.001						(Sandborn et al., 2020)

Drug	Study phase	Induction treatment				Study type	Maintenance treatment				References
		Week	Study drug	Placebo	*p*		Week	Study drug	Placebo	*p*	
b) UC											
Ustekinumab^a^	III	8	15.5	5.3%	<0.001	rr	52	43.8	24.0%	<0.001	Sands et al. (2019b)
Mirikizumab	II	12	22.6	4.8%	0.004						Sandborn et al. (2020b)

NB: In studies that feature different dosages or intervals, the most effective treatment has been presented.

^a^currently licensed for use as therapy in CD and UC; OLE, open-label extension; tt, treat-through; r, continuation of assigned treatment in responders; r 26, remission at week 26; rr, randomized responder; nd, not done; na, not applicable.

## Comment

Taken together, results from the aforementioned studies suggest that combined inhibition of both IL-12 and IL-23 (as achieved with ustekinumab and possibly briakinumab) as well as selective inhibition of IL-23 alone (with brazikumab, risankizumab, mirikizumab, and guselkumab) are all effective therapies for patients with CD, and probably also for those with UC. It is not yet clear whether inhibition of one vs*.* two of the target cytokines offers advantages with regard to effectiveness and/or safety. In particular, ustekinumab, which has been approved for use as a treatment for both CD and UC, has a documented favorable safety profile based on the results of individual studies of patients who received this therapy for more than 5 years. We can assume that this good safety profile will most likely apply to the other, related drugs considered here, although their individual adverse event profiles require specific evaluation in future studies.

## JAK Inhibitors

The therapeutic modalities considered above were designed to interact with extracellular targets, including pro-inflammatory cytokines or cell-adhesion molecules. Another concept under development involves the blockade of intracellular signaling using inhibitors of tyrosine kinases. While more than 80 tyrosine kinases have been characterized, the Janus kinases (JAKs) have been identified as critical targets. Tofacitinib is a small-molecule inhibitor of JAK1 and JAK3 that has already been approved for the treatment of UC. A clinically relevant advantage of this class of medications is that unlike biologics, they are largely non-immunogenic. This is an important attribute, given that neutralizing immune responses are often the cause of secondary loss of response to biologics [see Overview (Vulliemoz et al., 2020)].

However, one notable disadvantage of tofacitinib is the increased risk for primary infection or reactivation of herpes zoster, as well as deep vein thrombosis and pulmonary embolism, especially in elder patients. **Upadacitinib**, a selective JAK1 inhibitor, has already been approved for the treatment of refractory rheumatoid arthritis and is currently under evaluation as induction and maintenance therapy for moderate to severe CD and UC. Clinical and endoscopic remissions after 16 weeks were defined as primary clinical end-points in these studies. The current results reveal endoscopic improvement and clinical responses to induction therapy at daily doses of 6 and 7.5 mg, respectively. Of significant clinical relevance, the results of this study revealed that extraintestinal manifestations improved more frequently in response to upadacitinib compared to placebo in patients with CD (Peyrin-Biroulet et al., 2019).

Interestingly, more infections and more serious infections occurred in patients with CD in the upadacitinib treatment group than were reported in the group receiving placebo. While this was largely expected, this was not observed in studies involving the treatment of patients with UC (Sandborn et al., 2020a; Sandborn et al., 2020c).

**Filgotinib** is a selective JAK1 inhibitor that has recently been approved in Europe for the treatment of rheumatoid arthritis (September 2020); it has also been evaluated in patients with CD in the FITZROY phase II trial. Clinical remission was observed after 10 weeks in 47% of the patients receiving the study drug, compared to 23% in the placebo group (*p* = 0.008) (Vermeire et al., 2017b). A phase III trial in patients with CD is currently ongoing, together with additional phase II trials that feature subgroups of CD patients, including those with perianal fistulas or primarily small bowel disease. The recently closed phase IIb/III SELECTION study examined 1,348 patients with moderate to severe UC. Although the full publication remains pending, preliminary data reveal a significantly higher rate of clinical remission after 10 and 58 weeks among patients treated with 200 mg of filgotinib compared to placebo.

The results of six studies focused on achieving clinical remission in both CD and UC using JAK inhibitors are summarized in Table 3.

**TABLE 3:** Frequency of achieving clinical remission in studies featuring JAK inhibitors in patients with CD (a) or UC (b).

Drug	Study phase	Induction treatment				Study type	Maintenance treatment				Ref
		Week	Study drug	Placebo	*p*		Week	Study drug	Placebo	*p*	
a) CD											
Tofacitinib^a^	II	8	43.5	36.7%	0.325	rr	26	55.8	38.1%	0.13	Panés et al. (2017)
Upadacitinib	II	16	27.0	11.0%	<0.1	rr	52	41.0	32.0%	n.s	Sandborn et al. (2020c)
Filgotinib	II	10	47.0	23.0%	0.008						Vermeire et al. (2017b)

Drug	Study phase	Induction treatment				Study type	Maintenance treatment				Ref
		Week	Study drug	Placebo	*p*		Week	Study drug	Placebo	*p*	
b) UC											
Tofacitinib^a^	III	8	17.6	6.0%	<0.001	rr	52	40.6	11.0%	<0.001	Sandborn et al. (2017)
Upadacitinib	II	8	19.6	0.0%	0.002						Sandborn et al. (2020c)
Filgotinib	II/III	10	18.2	9.7%	0.001	rr	58	37.2	11.2%	<0.001	Feagan et al. (2020b), pooled data, Peyrin-Biroulet et al. (2020b)

NB: In studies that feature different dosages or intervals, the most effective treatment has been presented.

^a^currently licensed for use as therapy in CD and UC; OLE, open-label extension; tt, treat-through, r, continuation of assigned treatment in responders; r 26, remission at week 26; rr, randomized responder; nd, not done, na, not applicable.

In addition to the previously characterized JAK inhibitors, other innovative approaches are currently under evaluation in clinical trials. For example, another potential therapeutic target is tyrosine kinase 2 (TYK2); this kinase is involved in the signal transduction mediated by the IBD-associated cytokines IL-12, IL-13, and the interferons. The TYK2 inhibitor, BMS-986165, is currently under evaluation for induction and maintenance of remission in a phase II trial in patients with CD.

Local/topical administration of JAK inhibitors is an important approach that is currently under consideration in an effort to reduce the frequency of treatment-associated infections. Toward this end, TD 1473 is a high-affinity JAK1-, JAK2-, JAK3- and TYK2-inhibitor that cannot be resorbed; this property results in high levels of drug within the inflamed gut mucosa with only minimal systemic exposure. Results of phase I trials indicated low plasma levels of this drug despite high concentrations in the colon. Data obtained in trials that included healthy subjects and also patients with UC revealed that a single dose of up to 1,000 mg or 14 days of a 300 mg daily dose appear to be safe and well-tolerated. As such, this might prove to be a promising approach for the treatment of UC.

## Comment

In the coming years, additional tyrosine kinase inhibitors will be available for the treatment of IBD. Before treatment, the risk of herpes zoster reactivation and postherpetic neuralgia should be assessed and susceptible patients should be vaccinated. Likewise, there is an urgent need for direct head-to-head studies that compare the therapeutic potential of JAK inhibitors with previously-approved biological therapies. Due to the complexity of the JAK-mediated intracellular signaling pathways, it will be necessary to establish intensive long-term monitoring of patients who participated in registry studies to identify any long-term sequelae, including the potential for malignancies.

## Modulation of Sphingosine-1-Phosphate Receptors

Naïve lymphocytes reach the lymph nodes via afferent lymphatic vessels and ultimately enter the systemic circulation in response to an S1P gradient. Ligand-mediated activation leads to S1P-receptor (S1PR) internalization and therefore to a functional blockade of lymphocyte migration.

**Fingolimod** is an S1PR1 modulator that has already been approved for the treatment of multiple sclerosis. However, the use of this medication is associated with severe adverse effects, including cardiac sequelae, varicella zoster-encephalitis, hepatopathies, macular edema, and progressive multifocal leukoencephalopathy. A recent “Direct Health Care Professional Communication” issued by the EMA focused on fingolimod-induced liver injury, which can progress to acute liver failure requiring a liver transplant.

The TOUCHSTONE study featured responses of patients with UC to 32 weeks of therapy with the S1PR1/R5-modulator, **ozanimod**. The results of this study revealed a higher rate of clinical remission among those treated with the higher dose (1 mg/day) but not the lower dose (0.5 mg/day) of drug vs. those receiving placebo; this response was accompanied by a higher rate of mucosal healing (Sandborn et al., 2016). Data from the prolonged follow-up study (currently available as an abstract only) suggest that long-term control of inflammation can be achieved for up to 200 weeks.

An abstract of the results of the phase III study (TRUE NORTH) was presented at UEG Week 2020. In this study, 645 patients with UC were randomized 2:1 to receive treatment with 1 mg ozanimod (1 mg/day vs. placebo). After 10 weeks of therapy, clinical remission was achieved in 18.4 vs*.* 6.0% (*p* = 0.001) of the study participants, respectively; clinical remission was associated with a higher rate of mucosal healing. In the maintenance study that followed, 37% of the patients, that initially responded to the induction therapy reached a clinical remission after 52 weeks, compared to only 18.5% of those receiving placebo (*p* < 0.001) (Danese et al., 2020b; Sandborn et al., 2020f).

Preliminary data indicate that ozanimod is also effective in patients with CD. Sixty-nine patients were treated with ozanimod in the open STEPSTONE trial; after 12 weeks, 56.5% had a clinical response, 39.1% experienced clinical remission, and 23.2% had an endoscopic response (Feagan et al., 2020a). A placebo-controlled, phase III trial has been initiated.

A phase II study of the efficacy of **etrasimod**, an S1PR1/R4/R5 receptor modulator, in patients with UC indicated that treatment with this drug resulted in a marked improvement in the modified Mayo Scores when compared to placebo; this was especially notable in response to the higher dose (2 mg/day). Furthermore, a larger proportion of patients who achieved in clinical remission (33.0 vs. 8.1%; *p* < 0.001) and endoscopic improvement (41.8 vs. 17.8%; *p* = 0.003) could be identified after 12 weeks (Sandborn et al., 2020d).

Moreover, data from CD patients are available from a proof-of-concept-study that featured the S1PR1-modulator, **amiselimod**, although no clinical responses or biochemical improvement of disease activity was observed in comparison to placebo.

The results of four studies focused on achieving clinical remission in both CD and UC using S1PR modulators are summarized in Table 4.

**TABLE 4:** Frequency of achieving clinical remission in studies featuring S1P-receptor modulators to treat patients with CD (a) or UC (b).

Drug	Study phase	Induction treatment				Study type	Maintenance treatment				Ref
		Week	Study drug (%)	Placebo	*p*		Week	Study drug	Placebo	*p*	
a) CD											
Ozanimod	II	12	39.1	nd	Na						Feagan et al. (2020a)
Amiselimod	II	12	28.2	40.5%	ns						D’Haens et al. (2019)

Drug	Study phase	Induction treatment				Study type	Maintenance treatment				Ref
		Week	Study drug	Placebo	*p*		Week	Study drug (%)	Placebo	*p*	
b) UC											
Ozanimod	III	10	18.4	6.0%	<0.001	rr	52	37.0	18.5%	<0.001	Danese et al. (2020a), Sandborn et al. (2020)
Etrasimod	II	12	33.0	8.1%	<0.001	tt	46	39.3	nd	Na	Vermeire et al. (2019), Sandborn et al. (2020d)

OLE, open-label extension; tt, treat-through; r, continuation of assigned treatment in responders; r 26, remission at week 26; rr, randomized responder; nd, not done; na, not applicable; ns not significant. NB: In studies that feature different dosages or intervals, the most effective treatment has been presented.

## Comment

As the modulation of the S1PRs is a novel therapeutic approach, its overall efficacy specifically for the treatment of patients with IBD requires further examination. Initial findings suggest no essential advantages when compared to previously established therapeutic regimens. Furthermore, it is unclear as to what might happen when the drug is discontinued. Of concern is the possibility of excessive rebound inflammation due to sudden removal of the blockade to lymphocyte migration.

## Phosphodiesterase Inhibitors

**Apremilast** is an inhibitor of the intracellular enzyme, phosphodiesterase 4. Blockade of this signaling pathway results in increased concentrations of intracellular cyclic AMP (cAMP); this leads to the inhibition of TNF-α−release and an increase in the level of the anti-inflammatory cytokine, IL-10. Apremilast has already been approved for the treatment of psoriasis. Although higher rates of clinical remission were observed at week 12 in comparison to placebo (30 mg: 31.6% and 40 mg 21.8 vs*.* Placebo: 12.1%; *p* = 0.269 and *p* = 0.01, resp.) in a current phase II study with 170 UC patients, these results did not achieve statistical significance (hierarchical, stepdown testing procedure), and as such, the primary endpoint of the study was not reached. However, remission was maintained in up to 40% of the patients that remained on the medication at week 52 (Danese et al., 2020b).

## Comment

Inhibition of TNF-α *via* a mechanism that is unlikely to be vulnerable to immune-mediated loss of response is an interesting new therapeutic approach. However, the efficacy of this modality remains to be verified in future studies.

## Oligonucleotide-Based Therapeutics

Specific inactivation of genes known to be involved in disease pathogenesis might constitute an effective therapeutic modality with fewer adverse effects. Clinical experience with this type of therapy is currently very limited. The phase III study featuring administration of **mongersen**, an antisense oligonucleotide inhibitor of Smad7, which is a critical regulator of TGF-ß mediated down-regulation of pro-inflammatory cytokines, did not fulfill the high expectations from the phase II study for patients with CD. The study was terminated early due to insufficient efficacy (Sands et al., 2020a).

The anti-sense oligonucleotide therapeutic, **alicaforsen,** leads to down-regulation of intercellular adhesion molecule-1 (ICAM-1). Although this agent had some impact on disease activity, no convincing efficacy was demonstrated in a phase II trial with patients diagnosed with IBD. There is an ongoing phase III trial in which this agent is administered *via* an enema preparation specifically in patients with pouchitis. At UEG Week 2020, the first findings released from a phase IIb study with **cobitolimod** used to treat patients with left-sided ulcerative colitis were presented. This oligonucleotide TLR 9-agonist induces the expression of both IL-10 and type I-interferon. Two topical administrations of 250 mg were well tolerated by 42 of the total 213 patients participating in the study. The initial findings also suggest superiority regarding clinical remission after 6 weeks. No long-term data are currently available, but a phase III study is planned.

## Comment

The Mongersen study is an impressive example of the fact that positive results from phase II trials, notably those that mainly rely on subjective criteria such as the Clinical disease Activity Index (CDAI) as endpoints, may ultimately fail to be reproduced in larger phase III studies that apply objective criteria (e.g., luminal inflammation markers, such as fecal calprotectin, and/or endoscopic changes.) Likewise, although the study examining the rectal administration of cobitolimod provided useful proof of efficacy, this route is not suitable for long-term therapy of chronic diseases. Other modes of application will need to be developed.

## Fecal Microbiota Transplantation: Ready for Prime Time?

An inappropriate immunologic response to the gastrointestinal microbiota is thought to be of great importance in the pathogenesis of IBD. Numerous clinical observations suggest a close connection between pathologic gastrointestinal microbiota and the manifestation of disease in patients with IBD. However, considering that genetic factors and inflammatory reactions can change the composition of the gastrointestinal microbiota, it is not clear whether changes in the gut microbiome are the causes or consequences of the disease process. While FMT may be among the more drastic of the interventions used to influence the gastrointestinal microbiome, this modality is now an accepted regimen for the treatment of recurrent *Clostridium difficile* infection (Khoruts et al., 2020).

Multiple case studies and several randomized trials have explored this concept in patients with IBD. While most randomized studies involving patients with UC revealed that FMT had a significant impact on induction of remission (Stallmach et al., 2019; Tan et al., 2020), its efficacy has not been verified for patients with CD. Placebo-controlled trials involving patients with UC revealed that, although one out of every 3 to 4 patients achieved remission after FMT, one-time or short-term FMT leads to clinical failure over time. To address this problem, repeat colonoscopic FMT was performed once every 8 weeks in patients with UC; this led to the maintenance of both endoscopic and histologic remission. The role of FMT for the treatment of CD has been explored in case series and a single randomized study (Tan et al., 2020). Despite the very heterogeneous approaches, including different applications, target criteria, and patient groups (children, teenagers, or adults with CD), the data suggest an ∼30% probability (76/255 patients) of remission in response to FMT. However, in the first and thus far only published controlled study, Sokol et al. (Sokol et al., 2020) reported no difference in the remission rates maintained at 24 weeks between the FMT and sham-control group (4/8 vs. 3/9). As such, proof of the efficacy of FMT in patients with CD remains pending.

## Comment

To induce long-term and sustainable changes in microbiota, repetitive FMT applications are required. Our data in patients with UC have revealed that the diversity of gastrointestinal microbiota increases significantly and remains stable in response to the daily application of FMT-capsules over a 3 month period. Before the therapeutic concept of FMT can be established as a routine treatment for UC, several critical questions remain to be answered:

• Are certain donors more likely to be effective than others? Are there patient-specific factors that might be used to inform donor selection?
• When should FMT be performed in patients diagnosed with IBD? At an early stage of the disease or after exhaustion of all other established treatments?
• What is the influence of emerging infectious disease challenges (e.g., the COVID-19 pandemic) on the long-term success of an FMT protocol?

Of course, identification of the therapeutically active substance or substances in fecal microbiota presents the possibility that they might be produced exogenously and provided by more routine therapeutic routes. Nevertheless, FMT is an extremely interesting therapeutic concept that merits further consideration. However, due to multiple limitations and many as-yet unanswered, this procedure should not be performed outside of clinical trials.

## Conclusion

This review covers the current status of seven different new therapeutic approaches that may be on the horizon for the treatment of IBD. The modalities shown to be effective in phase II studies of course require confirmation in larger phase III studies; in most cases, these trials are already underway. The approval of several new therapeutic agents is anticipated in the near future, including many with unique pharmacological mechanisms of action. By their nature, these novel therapeutics will enhance and broaden the scope of our currently available drug treatments for IBD. This of course leads to further questions as clinicians attempt to determine which therapeutic approach is the best option for each patient. Given the broad scope of disease manifestations, it is unlikely that all patients will benefit equally from each new drug or drug class. One important focus of future studies will be the identification of biomarkers that can be used to predict individual responses to therapy. Until such predictors are available, the responsible clinician focusing on personalized therapy will have the option to select a given therapy based on individual disease characteristics, treatment targets, and published literature.
